# The oblique triangle configuration of three parallel screws for femoral neck fracture fixation using computer-aided design modules

**DOI:** 10.1038/s41598-021-03666-1

**Published:** 2022-01-10

**Authors:** Ru Yi Zhang, Jian Tao Li, Jing Xin Zhao, Zhe Zhao, Li Cheng Zhang, Cai Yun, Xiu Yun Su, Pei Fu Tang

**Affiliations:** 1 Department of Orthopaedics, Beijing Shijingshan Hospital, Shijingshan Teaching Hospital of Capital Medical University, No. 24, Shijingshan Road, Beijing, 100043 China; 2 Department of orthopedics, The fourth medical center, General Hospital of Chinese PLA, Beijing, China; 3 National Clinical Research Center for Orthopaedics, Sports Medicine and Rehabilitation, Beijing, 100853 China; 4 Department of Orthopaedics, School of Clinical Medicine, Beijing Tsinghua Changgung Hospital, Tsinghua University, Beijing, 102218 China; 5 Department of Orthopaedics, Zouthern University of Science and Technology Hospital, Shenzhen, 518055 Guangdong China

**Keywords:** Musculoskeletal system, Trauma

## Abstract

Closed reduction and internal fixation with three cannulated compression screws is a common method for treating femoral neck fractures in young and middle-aged patients. Protocols including the inverted triangle configuration and dispersion of the screws still needed further supports. The purpose of this study was to explore a novel oblique triangle configuration (OTC) of three screws in fixing femoral neck fractures based on the morphology of the femoral neck isthmus (FNI). The computer-aided design modules were used to explore the ideal spatial configuration with largest triangle by three parallel screws. A univariate evaluation model was established based on the oval-like cross-section of the FNI. When the three screws were positioned by the OTC, Inverted Equilateral Triangle Configuration (IETC), and the Maximum Area Inverted Isosceles Triangle Configuration (MA-IITC) respectively, the proportion of area and circumference in the cross-section of FNI and the changing trend of proportion were compared under various torsion angles, eccentricity, and cross-sectional area of FNI. The area and circumference ratios of the parallel screws using the OTC method were significantly higher than in the IETC and MA-IITC groups. In the univariate evaluation model, the OTC area ratio and circumference ratio remained stable under the different femoral neck torsion angles, FNI cross-sectional area, and eccentricity. The OTC method provided an ideal spatial configuration for the FNA fixation with the largest area using three parallel screws. The position of the posterior screw was also away from the metaphyseal artery, potentially reducing the possibility of vascular injury and screw penetrating.

## Introduction

Femoral neck fracture is a common fracture of the lower limbs due to high-energy injuries in young and middle-aged patients^1,2^. Because of the limited blood supply of femoral neck, it is prone to avascular necrosis of femoral head and nonunion after improper internal fixation^3,4^. Multiple studies have demonstrated that the percutaneous, minimally invasive fixation of femoral neck fractures using cannulated compression screws (CCSs) remained the preferred choice in terms of the lower displacement rate^4,5^. The parallel screw fixation is currently a common choice in treating femoral neck fractures^4,6,7^. Furthermore, techniques included the inverted triangle, cortical support, and separation have been proposed to improve the stability of internal fixation and reduce the risk of nonunion and necrosis postoperatively^8,9^. Parallel implant and cortical support were proved feasibly to achieve using computer navigation technology^10,11^. However, the inverted triangle pattern and separated technics were controversial since there was no specific method to evaluate the outcomes.

The morphology of the femoral neck isthmus (FNI) has a significant impact on the spatial configuration of the implanted screws when performing the internal fixation of the femoral neck fracture. The biomechanical analysis also proved that the different alignment or dispersion of the screws had a significant influence on the stability of the internal fixation^12^. Anatomical studies had shown that the cross-section morphology of the femoral neck isthmus was approximately oblique elliptical and affected by the torsion angle, cross-sectional area, and eccentricity of the femoral neck^13,14^. This study was aimed to explore the optimal spatial configuration of the cannulated three parallel screws for femoral neck fracture in the principles of cortical support and the maximum area of the inscribed triangle ellipse^15–17^. Under the computer-aided model, the area and perimeter of the novel configuration (OTC) were compared with the group of traditional inverted isosceles triangle configuration (IETC) and equilateral triangle configuration. The tendency of the area and circumference ratio of different triangular configurations was analyzed in various torsion angles, cross-sectional areas, and eccentricities of ellipse models.

## Materials and methods

This study was performed after obtaining the approval of the Institutional Review Board (IRB) of the General Hospital of the Chinese People’s Liberation Army. A torsional ellipse model was established using the AutoCAD 2007 software (Autodesk Inc., America) to assess the properties of various configuration models^13^. In order to reduce the individual bias, the morphological data of the FNI cross-section applied in the configuration models was based on our previous study of 200 cases of femoral neck morphology variations^14,18^. The parameters affecting the morphology of the femoral neck were set in this torsional ellipse model and listed as follows: the torsion angle of the ellipse was 30°, the long and short axis was 17.50 mm and 14.00 mm, and the eccentricity was 0.60^18^.

In this study, the femoral calcar screw (FCS) was first implanted according to the protocol of parallel inverted triangles. Because of the anatomical torsion of the femoral neck, two operating models with and without torsion angle variables were performed. On the cross-section of FNI, the FCS was first implanted at the bottom of the femoral neck cavity on anteroposterior X-ray and the centerline of the femoral neck on lateral X-ray. The second model was that the FCS was implanted at the lowest point of the femoral neck. Because of the existence of the femoral neck torsion, the screw in the second model was positioned backward relative to the centerline of the femoral neck on lateral X-ray.

**Figure 1:**
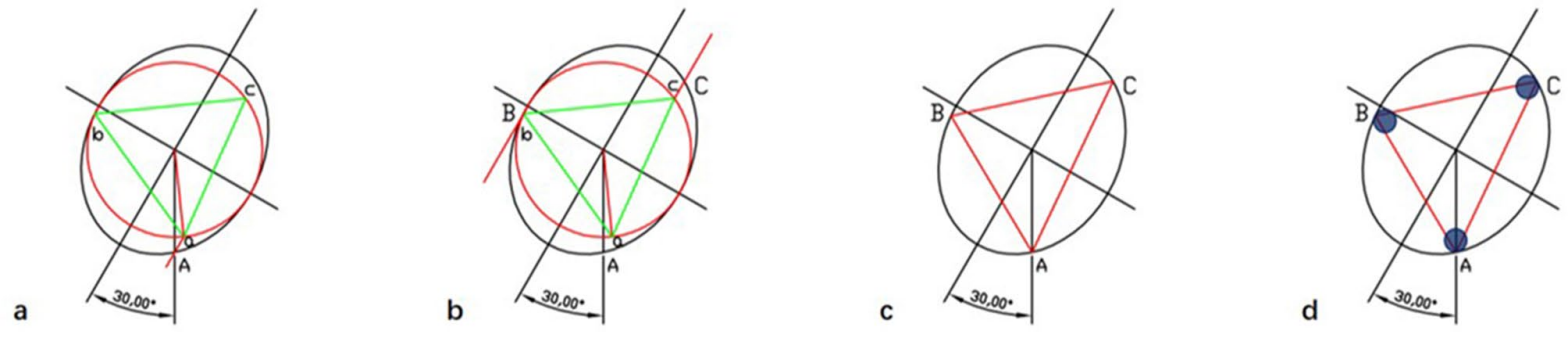
An oblique ellipse model was established based on the morphological features of the cross-section of the femoral neck isthmus. The screws on the ellipse were based on the cortex support of the femoral neck. An oblique triangle was drawn on the ellipse according to the method of inscribed triangles with the largest area of the ellipse. The predicted insertion of the femoral calcar screw is defined as point A, which is implanted at the bottom of the femoral neck cavity on anteroposterior X-ray and the centerline of the femoral neck on lateral X-ray. The oblique triangle was drawn as follows: (**a**) Point A was determined by the intersection of the vertical lines through the ellipse center and the ellipse, which was the position of the first screw in the OTC method. A line was drawn parallel to the long axis of the ellipse through point A and the intersection of the line and the inscribed circle was defined as point a. The largest inscribed triangle (isosceles triangle) of the circle was made with point a as the vertex, and the other two vertices were then defined as point b and point c. (**b**) Two lines through points b and c were drawn parallel to the long axis of the ellipse, and the intersection points of the two lines and ellipse were set as the position of the screws (points B and C). (**c**) The triangle was formed by three points of A, B, C, which was the inscribed triangle (oblique triangle) with the largest area. (**d**) Screws position in oblique triangle configuration (blue dot).

In the torsional ellipse model, we first determine the cross-section of the femoral neck isthmus. The largest inscribed circle in the ellipse was drawn, and an equilateral triangle was inscribed in the circle, the endpoints were marked as “*a, b, c*”. Point A (position of the femoral calcar screw) was determined parallel to the major axes of the ellipse as an endpoint of the oblique triangle. The other two points are made on the ellipse according to the endpoint “*b, c*” parallel to the major and minor axes of the ellipse, forming an inscribed triangle on the ellipse (Fig. 1)^16,19^. The largest inscribed triangle on the ellipse was approached under the CAD model.

**Figure 2:**
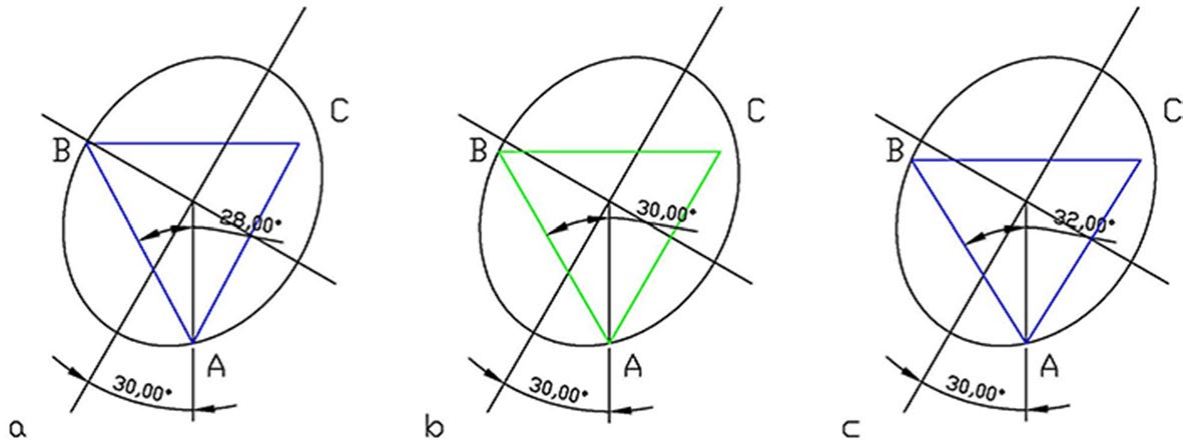
By adjusting the angle between the line connecting point A and point B and the line connecting point A and the center of the ellipse, the area of the largest triangle was gradually approached (ranged from 28° to 32°). The largest area of the isosceles triangles was selected as the inverted isosceles triangle configuration.

Then, according to the IETC method, the position of the front and posterior screws on the ellipse were determined. And then, the position of the point B was moved in the ellipse and gradually approached the optimal position of the front and posterior screws with the largest IITC area (Fig. 2). Finally, the triangle area and perimeter of the three configurations were measured.

**Figure 3:**
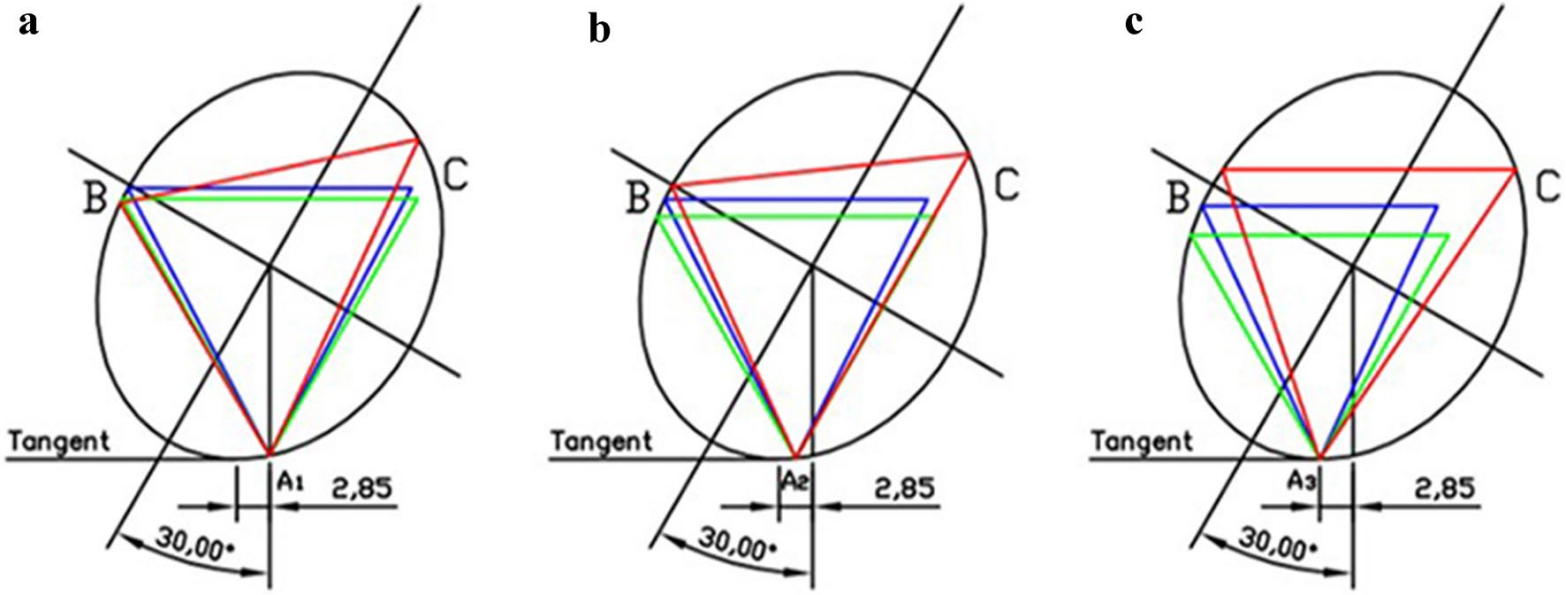
In the ellipse model, when the femoral calcar screws were positioned at different points (A1, A2, A3), there were three triangle configurations. The red triangle is OT; the blue is MA-IIT; the green is IET. (**a**) The femoral calcar screw is located at point A1 below the femoral neck; (**b**) the femoral calcar screw was located at point A2, and the midpoint of point A1 and A3; (**c**) the femoral calcar screw was located at point A3: The lowest point of the femoral neck in the anterior and posterior position. At this time, the screw was not located at the midline of the femoral neck cross-section while was located backward relative to the vertical line.

We set the position of the femoral screws in two operation models as A1 and A3 points respectively and define the midpoint A2 between A1 and A3 on the ellipse. We fixed these vertices to determine the other two vertices B and C, respectively, by the method of inscribing a triangle with the largest area on the ellipse (Fig. 3). The area and perimeter among the Oblique Triangle (OT), Inverted Equilateral Triangle (IET), and Maximum Area Inverted Isosceles Triangle (MA-IIT) were measured.

Under the univariate evaluation model (variables included the torsional angle, area, and eccentricity), a torsional ellipse was first used to simulate the cross-sectional shape of the femoral neck isthmus^13^. The torsional angle of the ellipse in the first model was set at 10°, 20°, 30°, 40°, and the area and eccentricity are fixed. The second model was to adjust the ellipse area (the semi-major axis was 12.5, 15, 17.5, and 20 mm), and the torsion angle and eccentricity remain the same. The third model was to adjust the ellipse eccentricity (0.4, 0.5, 0.6, 0.7). The largest inscribed triangle (OT), IET, and MA-IIT of the known fixed vertices in the ellipse, and the area and perimeter of these three triangles were drawn. The area and perimeter of the three triangles, and the proportion of the area and perimeter of the ellipse in the three models were compared.

## Results

**Figure 4:**
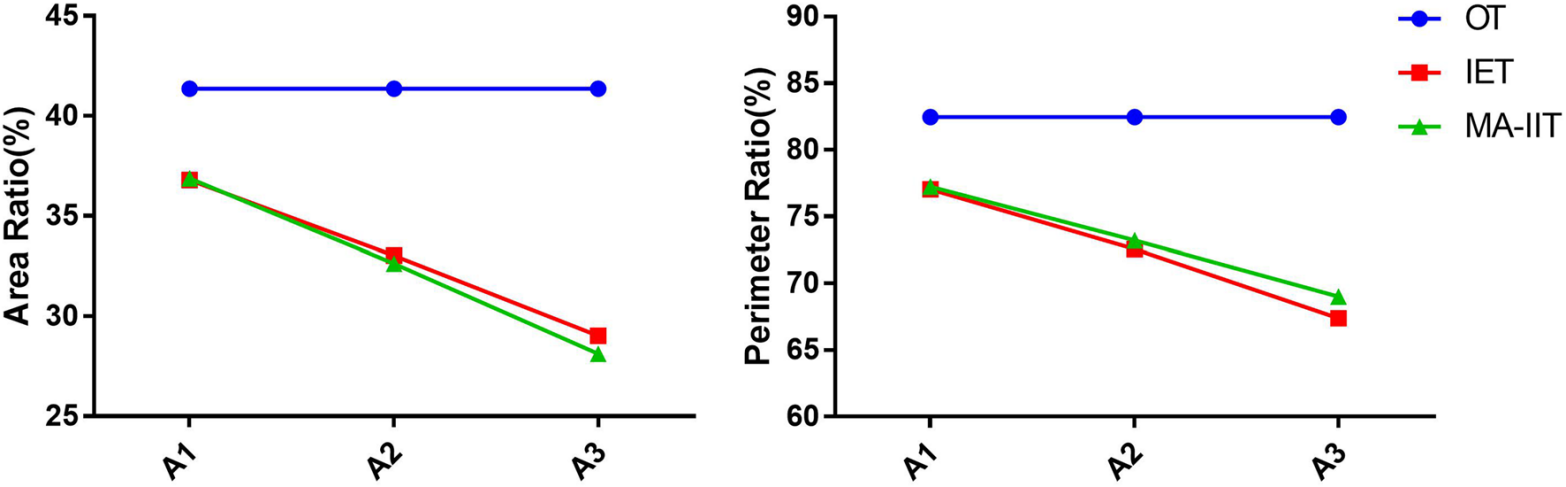
When the femoral calcar screw was located at different positions, with the clockwise change of point A, the trend of the area and circumference ratio of OT, IET, and MA-IIT in the ellipse.

**Figure 5:**
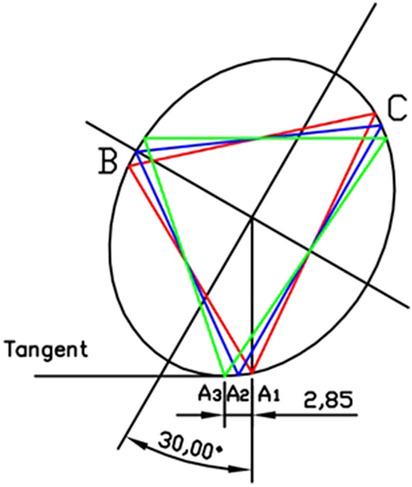
The position of the OT when the femoral calcar screw was located at different points (A1, A2, A3). The triangle with the femoral calcar screw at point A1 was red; the triangle with the femoral calcar screw at point A2 was blue, the triangle of the femoral calcar screw at point A3 was green.

Based on the ellipse model established in this study, the mean area and circumference of the ellipse were 769.69 mm^2^ and 99.57 mm. The area and perimeter proportions of OT, IET, and MA-IIT using the points A1, A2, and A3 as vertices in the ellipse were shown in Fig. 4. And the results show that the values of OT were significantly greater than IET and MA-IIT, and there was little difference between IET and MA-IIT groups. The area and perimeter of OT in this model remained stable with the position change of point A (A1, A2, A3), the area and perimeter of IET and MA-IIT gradually decrease when point A moves from front to back (Fig. 4). With point A moves, the position of the other two vertices changed in the same direction. And the triangle area moved according to the clockwise pattern (ellipse inclined to the right) (Fig. 5).

**Figure 6:**
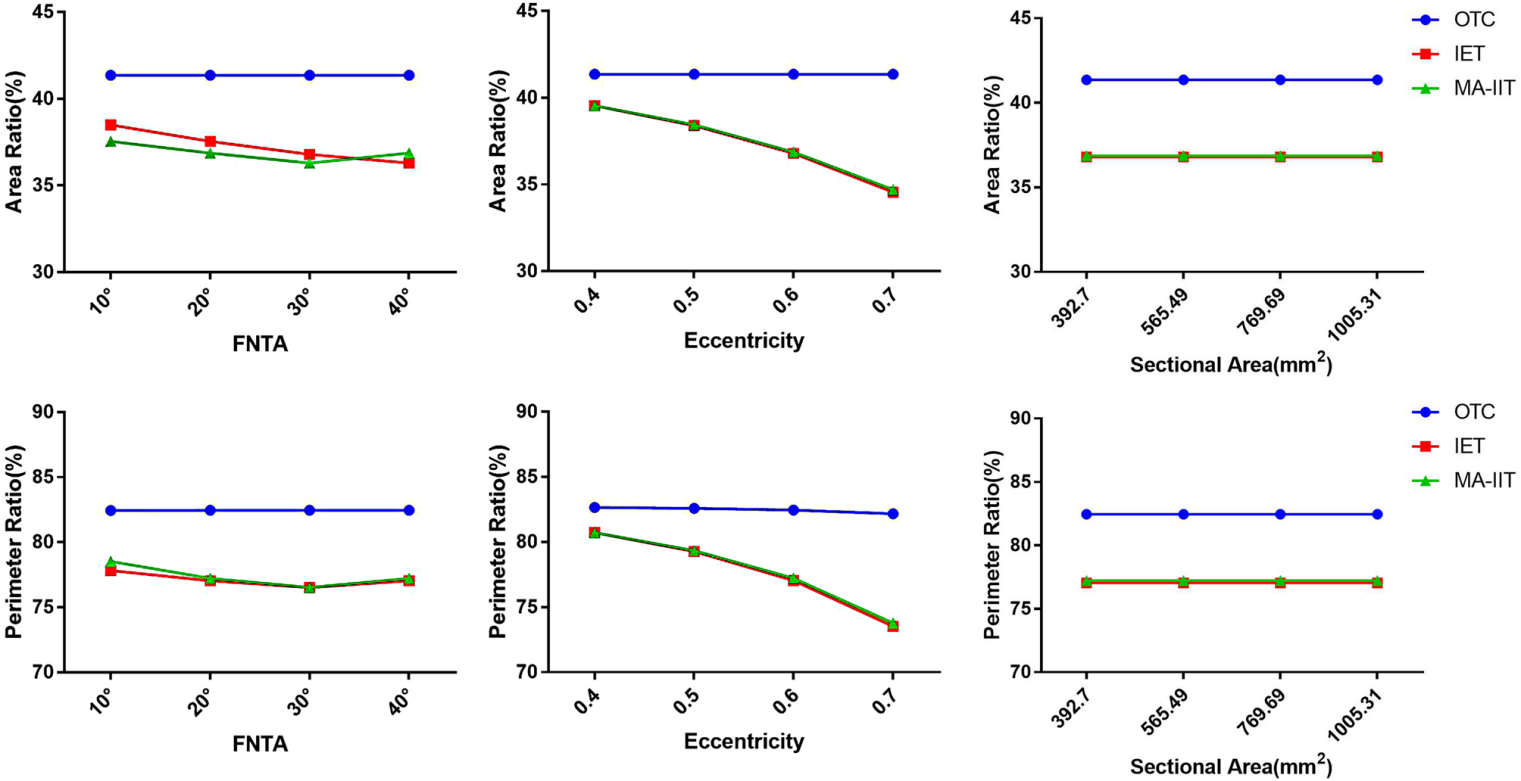
In the univariate evaluation model, the area and perimeter ratio of OT, IET, and MA-IIT under different variables. The area and perimeter ratio of OTC were stable under the given variables (torsional angle, area, and eccentricity) in the torsional ellipse model. The area ratio and circumference ratio of IET and MA-IIT decreased with the increase of the torsion angle and eccentricity of the ellipse, while parameters of OT were not affected by the area of the ellipse.

In the univariate evaluation model, the area ratio and perimeter ratio of OT, IET, and MA-IIT in the ellipse model was shown in Fig. 6. The area and perimeter ratio of OTC was stable under the given variables (torsional angle, area, and eccentricity) in the torsional ellipse model. The area ratio and circumference ratio of IET and MA-IIT decreased with the increase of the torsion angle and eccentricity of the ellipse, while parameters of OT were not affected by the area of the ellipse (Fig. 6). Moreover, there was little difference between IET and MA-IIT in both the area ratio and circumference ratio.

## Discussion

The data from this study proved that the parallel screws using oblique triangle configuration can obtain maximum inscribed area on the FNI cross-section compared with the other methods. The area and circumference of the OTC were significantly larger than traditional IET and MA-IIT configuration and may reduce the potential risks of traditional IET and MA-IIT configuration caused by the cortex penetrating and insufficient cortical support of the screws^19–21^.

Biomechanical and clinical studies have revealed that internal fixation with the parallel inverted triangle, large dispersion, and cortical support showed better mechanical stability and clinical outcomes^22–26^. The classic screw configuration was based on the rounded cross-section of the femoral neck. However, the cross-section of femoral neck is reported approximately elliptical in the normal population^27^. In this study, we constructed an oblique elliptical femoral neck isthmus using CAD software to simulate the structure of the femoral neck and used mathematical methods to prove that the OTC with parallel screws showed theoretical advantages in the fixation of femoral neck fractures. And it is more in line with the principles of screw placement: parallel, inverted triangle, dispersion, cortical support.

Lindequist et al. proved that the optimal support of the femoral neck cortex can be achieved when the distance between the screws and the femoral neck cortex was less than 3 mm^15^. This study found that the area and circumference parameters of the three screws show the lowest change rate during the position change in the OT configuration. While the area and circumference of IET and MA-IIT decreased with the position of the femoral calcar screw from front to back. As the position of the femoral calcar screw changes (clockwise), the positions of the other two screws also change in the clockwise direction. When the femoral calcar screw was implanted on the midline of the femoral neck on the lateral image, the position of the posterior superior screw would be far away from the posterior and superior femoral neck cortex. The position of the inferior screw was reported related to the incidence of stress subtrochanteric fracture, and the probability of stress subtrochanteric fracture may be higher when it is positioned below the level of the lesser trochanter^17,28^. Compared to the equilateral triangle configuration, the OTC and inverted triangle configuration with a single inferior screw may reduce the potential risk of subtrochanteric fractures. Meanwhile, the inferior screw needs to avoid being excessive inferiorly placed, preventing the penetration of cortical bone.

The screw distance and the triangle circumference of configuration were commonly used to evaluate the degree of screw dispersion^19,29^. The overall cross-sectional area of the internal fixation is positively related to the bending section modulus and the torsion section modulus^16,30^. The larger the overall cross-sectional area of the internal fixation indicated the more dispersed stress of the screws in the femoral head, and the less displacement of the fractured end^31^. The results of this study showed that the area ratio of OT configuration was not affected by the three factors of the inclined ellipse, and remains a stable value (0.413). Therefore, the three parallel screws with oblique triangle configuration showed the largest dispersion and were supposed to be an ideal screw space configuration in the internal fixation of femoral neck fractures.

The main branches of the superior, inferior, and anterior epiphyseal arteries in the epiphysis formed an approximately triangle arterial network^32,33^. When using an oblique triangle configuration, screws pass through the hypovascular area of the femoral neck, reducing potential iatrogenic injury of the epiphyseal artery. The inferior retinacular artery (IRA) is the main residual artery post-fracture, especially in displaced type. The IRA passes through the femoral neck between the area of the lesser trochanter and quadrate tubercle and subsequently enters the femoral head at the posteromedial area of the femoral neck^32^. When the femoral calcar screw was implanted on the midline of the femoral neck under the lateral X-ray fluoroscopy, the screw would be far away from the posteromedial area of femoral neck cortex, reducing the incidence of penetration of the screw and the IRA injury.

**Figure 7:**
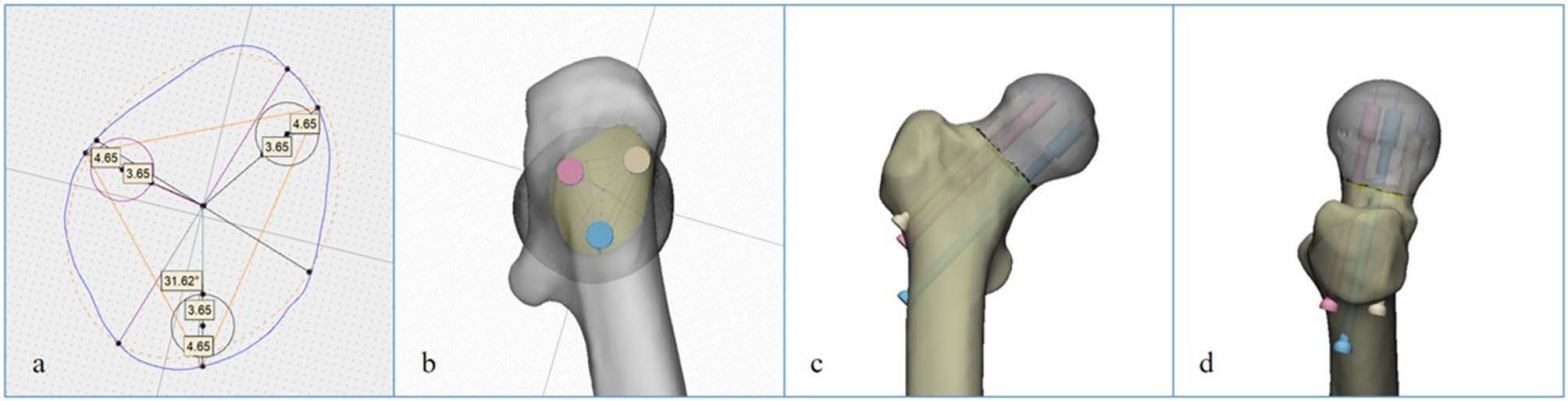
According to the oblique triangle configuration, the position of the three screws on the cross-section of the femoral neck isthmus by the 3-Matic software. (**a**) The blue line was the contour of the femoral neck isthmus, and the yellow dashed line was the fitted ellipse of the femoral neck isthmus. (**b**) The configuration of the screws according to the cross-section of the FNI. (**c**) the OTC in anteroposterior view, (**d**) the OTC in lateral view.

Tang et al. proposed the “triangular stability theory” of the proximal femur and pointed out that the proximal femur is stabilized by a structural mechanical model formed by the medial, lateral, and upper sides^26,34^. The medial side forms the oblique support of the cantilever configuration of the proximal femur, which greatly reduces the bending stress and deflection of the structure. The lateral side could significantly reduce the sliding and deflection of the femoral neck under physiological load, and achieve the stability of the fracture end. The upper side connects the medial and the lateral side which could resist the bending moment generated by the physiological load. When the femoral neck fractures, the upper and medial edges of the proximal femur were destroyed at once. The medial edge structure relies on the medial support of the femoral calcar screw to achieve reconstruction. The upper two screws in the oblique triangle configuration provide the best mechanical strength, reconstruct the upper side structure of the proximal femur, and significantly improve the mechanical stability of the fractured end. The configuration of three screws in the OTC model of femoral neck fracture was established to reduce the strain in the internal fixation component, maintain the stability of the fracture end, and promote fracture healing (Fig. 7).

This study has limitations. First, the univariate calculation model using the FNI cross-section provided an idealized experimental condition. Meanwhile, further study on the reconstruction of the entire cavity of femoral neck is required to better simulate the biomechanical stability of screw configuration. Second, parameters included the circumference and cross-section area were reported positively related to rotational stability of the internal fixation. While there was little clinical evidence in evaluating the relationship between these parameters and postoperative stability, which also requires further research to verify the association between the various triangle configuration and postoperative outcomes.

In conclusion, the OTC method provided an ideal spatial configuration with the largest area using three parallel screws for the FNA fixation. The position of the posterior femoral calcar screw was implanted far away from the metaphyseal artery, potentially reducing the possibility of vascular injury and screw penetrating. The OTC method might have potential advantages in mechanical properties in FNF fixation when obtaining the largest triangular area by three screws, while the effectiveness remains biomechanical studies to verify before clinical application.
